# Supplementary material for: Selenium Alleviates Cadmium Toxicity in Pepper (Capsicum annuum L.) by Reducing Accumulation, Enhancing Stress Resistance, and Promoting Growth
Source: Plants (Basel). 2025 Apr 24;14(9):1291. doi: 10.3390/plants14091291 (PMC12073608; doi:10.3390/plants14091291)
Supplement: Supplementary file 1 [file plants-14-01291-s001.zip › plants-3591144-supplementary.pdf]

Supplementary information:

Figure legends:

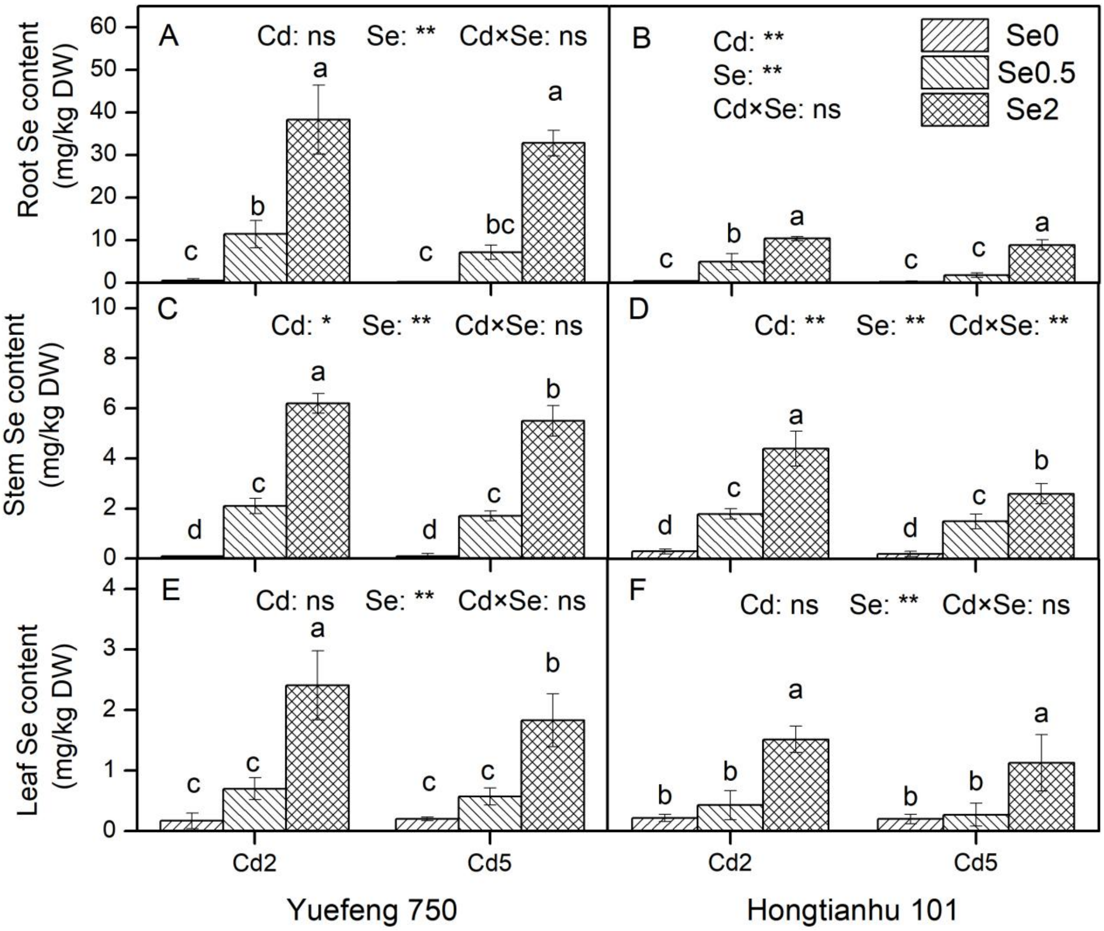

Figure S1. Effects of adding Cd and Se to nutrient solution on Se content in roots, stems and leaves of two pepper seedlings. Cd2 and Cd5 represent Cd content of 2 and 5 μmol/L in the nutrient solution, and Se0, Se0.5 and Se2 represent Se content of 0, 0.5, and 2 μmol/L in the nutrient solution, respectively. Changes in Se content in the roots (A, B), stems (C, D), and leaf (E, F) of Yuefeng 750 and Hongtianhu 101 under combined Cd + Se treatment. The mean values (±SD, n=3) followed by different lowercase letters indicates significant differences among different treatments ($P < 0.05$) according to two-way ANOVA followed by LSD test at the same variety. Significant main and interactive effects are indicated by * ($P < 0.05$) and ** ($P < 0.01$). ns indicates non-significant effects.

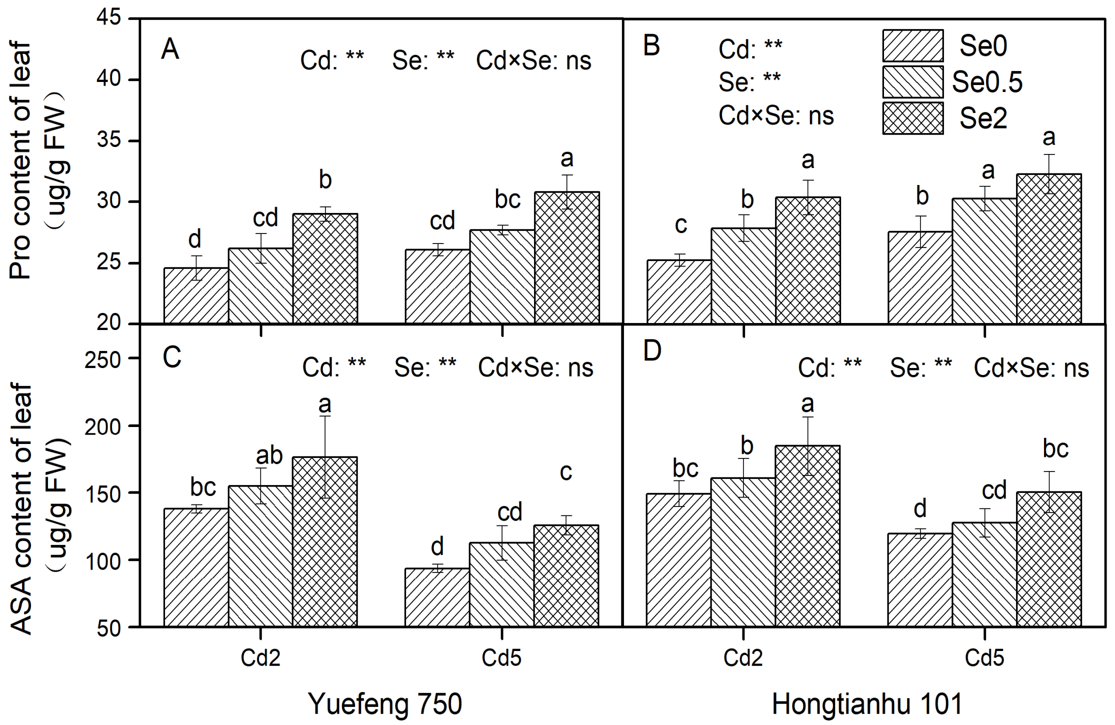

Figure S2. Effects of adding Cd and Se to nutrient solution on Pro and ASA content of leaves of two pepper seedlings. Cd2 and Cd5 represent Cd content of 2 and 5 μmol/L in the nutrient solution, and Se0, Se0.5 and Se2 represent Se content of 0, 0.5, and 2 μmol/L in the nutrient solution, respectively. Changes in the leaf Pro contents (A,B), and ASA contents (C,D) of Yuefeng 750 and Hongtianhu 101 under combined Se+Cd treatment. The mean values (±SD, n=3) followed by different lowercase letters indicates significant differences among different treatments ($P < 0.05$) according to two-way ANOVA followed by LSD test at the same variety. Significant main and interactive effects are indicated by * ($P < 0.05$) and ** ($P < 0.01$). ns indicates non-significant effects.

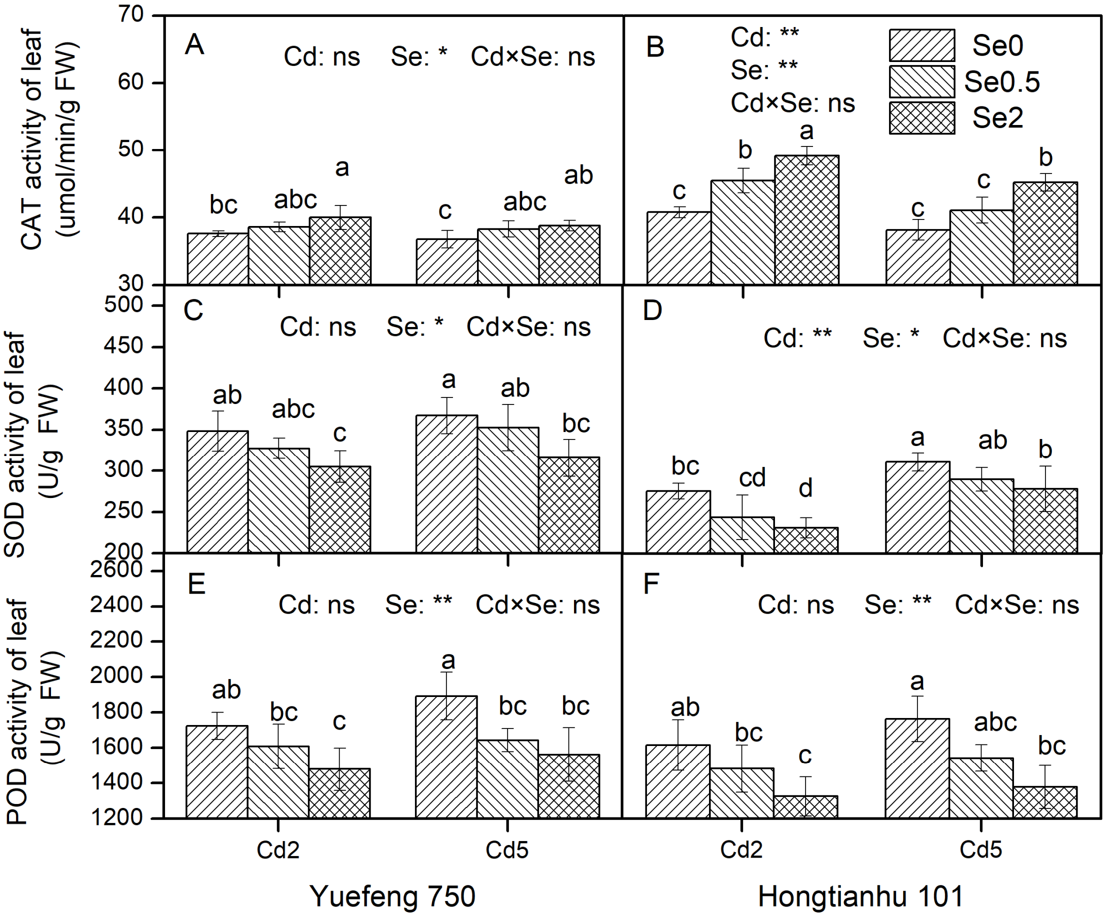

Figure S3. Effects of adding Cd and Se to nutrient solution on CAT, SOD, POD activity of leaves of two pepper seedlings. Cd2 and Cd5 represent Cd content of 2 and 5 μmol/L in the nutrient solution, and Se0, Se0.5 and Se2 represent Se content of 0, 0.5, and 2 μmol/L in the nutrient solution, respectively. Changes in the leaf CAT activity (A,B), SOD activity (C,D), and POD activity (E,F) of Yuefeng 750 and Hongtianhu 101 under combined Se+Cd treatment. The mean values (±SD, n=3) followed by different lowercase letters indicates significant differences among different treatments ($P < 0.05$) according to two-way ANOVA followed by LSD test at the same variety. Significant main and interactive effects are indicated by * ($P < 0.05$) and ** ($P < 0.01$). ns indicates non-significant effects.

Table legends:

Table S1. Effects of adding Cd and Se to nutrient solution on root morphological parameters and vitality of two pepper seedlings.

Variety	Treatment	Root morphological parameters					Root vitality [mg/(g·h)]
		Total root length (cm/plant)	Total root surface area (cm^2 /plant)	Total root volume (cm^3/plant)	Average root diameters (mm/plant)	Root tips number (per plant)	
Yuefeng 750	Cd2 Se0	423.2±19.8b	205.0±14.6bc	23.8±2.5bc	1.43±0.08ab	171.0±11.4b	11.5±1.4cd
	Cd2 Se0.5	479.0±14.7ab	216.0±18.9ab	26.2±1.6ab	1.35±0.11b	203.7±13.6a	17.3±2.6c
	Cd2 Se2	517.2±19.4a	242.3±12.8a	28.1±1.4a	1.33±0.07b	185.0±18.7ab	34.2±4.8a
	Cd5 Se0	325.6±79.8c	174.7±28.9c	22.3±0.9c	1.54±0.07a	132.0±20.9c	5.7±2.8d
	Cd5 Se0.5	405.6±31.2b	196.6±16.7bc	25.1±1.8abc	1.49±0.09ab	185.0±17.5ab	14.6±4.3c
	Cd5 Se2	427.0±27.7b	206.4±23.6bc	26.0±1.8ab	1.44±0.05ab	158.7±23.3bc	27.7±5.7b
Hongtianhu 101	Cd2 Se0	369.5±24.8c	167.3±8.1bc	23.1±1.3bc	1.31±0.06bc	153.0±16.4b	23.6±4.4de
	Cd2 Se0.5	456.1±12.8b	200.1±11.2ab	25.5±2.0ab	1.24±0.13c	185.7±11.4a	37.4±5.7bc
	Cd2 Se2	509.1±14.4a	221.7±10.5a	27.3±1.5a	1.20±0.07c	164.7±8.5ab	47.6±3.9a
	Cd5 Se0	194.1±28.7d	138.2±32.1c	21.6±1.4c	1.52±0.17a	103.0±17.0c	20.6±2.4e
	Cd5 Se0.5	351.3±27.7c	173.1±21.4bc	23.9±1.9bc	1.44±0.08ab	156.7±15.0b	30.7±4.1cd
	Cd5 Se2	380.2±16.2c	169.7±24.3bc	24.8±1.5ab	1.41±0.04ab	149.0±16.8b	38.8±3.1b
Statistical significance							
Variety（V）		**	**	ns	*	**	**
Cd		**	**	**	**	**	**
Se		**	**	**	*	**	**
V×Cd		*	ns	ns	ns	ns	ns
V×Se		*	ns	ns	ns	ns	ns
Cd×Se		ns	ns	ns	ns	ns	ns
V×Cd×Se		ns	ns	ns	ns	ns	ns

Note: The pepper seedlings were grown in nutrient solution with Cd (2 and 5 μmol/L) or/and Se (0, 0.5, and 2 μmol/L) for 26 d. The mean values (±SD, n=3) in each column followed by different lowercase letters indicates significant differences among different treatments ($P < 0.05$) according three-way ANOVA followed by LSD test at the same variety. Significant main and interactive effects are indicated by * ($P < 0.05$) and ** ($P < 0.01$). ns indicates non-significant effects.

Table S2. Effects of adding Cd and Se to nutrient solution on plant height, stem diameter and canopy width of two pepper seedlings.

Variety	Treatment	Plant height (cm)	Stem diameter (mm)	Canopy wide (cm)
Yuefeng 750	Cd2 Se0	17.1±1.1b	3.6±0.2b	23.4±1.8b
	Cd2 Se0.5	17.7±1.3b	3.7±0.3ab	24.7±1.1a
	Cd2 Se2	18.6±1.4a	3.9±0.2a	25.3±1.7a
	Cd5 Se0	14.1±1.9d	3.3±0.3c	21.5±2.4c
	Cd5 Se0.5	15.2±0.8c	3.5±0.3b	22.4±1.6bc
	Cd5 Se2	15.9±1.2c	3.6±0.1b	23.0±1.4b
Hongtianhu 101	Cd2 Se0	9.8±0.8c	3.0±0.4bc	19.5±1.4b
	Cd2 Se0.5	12.7±1.0b	3.2±0.3ab	22.6±1.4a
	Cd2 Se2	14.1±1.1a	3.4±0.3a	23.7±1.1a
	Cd5 Se0	8.4±0.8e	2.6±0.3d	16.8±1.7c
	Cd5 Se0.5	9.0±0.9de	2.8±0.3cd	17.4±1.5c
	Cd5 Se2	9.5±0.7cd	2.9±0.2c	18.6±1.5b
Statistical significance				
Variety（V）		**	**	**
Cd		**	**	**
Se		**	**	**
V×Cd		ns	*	**
V×Se		*	ns	ns
Cd×Se		**	ns	*
V×Cd×Se		**	ns	ns

Note: The pepper seedlings were grown in nutrient solution with Cd (2 and 5 μmol/L) or/and Se (0, 0.5, and 2 μmol/L) for 26 d. The mean values (±SD, n=3) in each column followed by different lowercase letters indicates significant differences among different treatments ($P < 0.05$) according three-way ANOVA followed by LSD test at the same variety. Significant main and interactive effects are indicated by * ($P < 0.05$) and ** ($P < 0.01$). ns indicates non-significant effects.
